# Free Fatty Acid Overload Targets Mitochondria: Gene Expression Analysis of Palmitic Acid-Treated Endothelial Cells

**DOI:** 10.3390/genes13101704

**Published:** 2022-09-22

**Authors:** Afraz Ahmad Raja, Abdullahi Dandare, Muhammad Jawad Khan, Muhammad Jadoon Khan

**Affiliations:** 1 Functional Genomics Laboratory, Department of Biosciences, COMSATS University Islamabad, Islamabad 45550, Pakistan; 2 Department of Biochemistry, Usmanu Danfodiyo University, Sokoto P.M.B. 2346, Nigeria

**Keywords:** gene expression, mitochondria, palmitic acid, free fatty acids, lipotoxicity

## Abstract

Lipotoxicity is known to cause cellular dysfunction and death in non-adipose tissue. A major cause of lipotoxicity is the accumulation of saturated free fatty acids (FFA). Palmitic acid (PA) is the most common saturated fatty acid found in the human body. Endothelial cells form the blood vessels and are the first non-adipose cells to encounter FFA in the bloodstream. FFA overload has a direct impact on metabolism, which is evident through the changes occurring in mitochondria. To study these changes, the PA-treated human coronary artery endothelial cell (HCAEC) dataset was obtained from the Gene Expression Omnibus (GEO), and it was analyzed to obtain differentially expressed genes (DEGs) from the nucleus and mitochondria. Functional and pathway enrichment analyses were performed on DEGs. Results showed that nuclear and mitochondrial DEGs were implicated in several processes, e.g., reactive oxygen species (ROS) production, mitochondrial fusion and fission, Ca^2+^ sequestering, membrane transport, the electron transport chain and the process of apoptosis. To better understand the role of FFA in endothelial cell damage, these DEGs can lead to future experiments based on these findings.

## 1. Introduction

Lipotoxicity is a condition in which a surplus of fatty acids in non-adipose tissue causes cellular dysfunction and eventually cell death [1]. This condition leads to different pathogeneses, and lipotoxicity has been linked to several complications, including obesity, a lack of insulin sensitivity, atherosclerosis and diabetes. Endothelial cells are the first cells that encounter the bloodstream; hence, they are an easy target for fatty acid overload [2]. Saturated and unsaturated fatty acids are known to have differential effects on cell death and survival, though the mechanisms associated with these differences are unknown [3]. Palmitic acid (PA) is the most abundant type of saturated fatty acid in the plasma [4]. It has been implicated in the toxicity of many different cell types, including pancreatic *β*-cells [5], hepatocytes [6] and several others [1,3]. Palmitic acid has also been reported to enhance cellular oxidative stress [7] and apoptosis [8]. In contrast, there is evidence that in the presence of unsaturated fatty acids, e.g., oleic acid, they promote autophagy but have minimal effects on apoptosis [8]. Oleic acid is known to form lipid droplets and it also directs PA to the droplets and lowers the amount of PA as FFA [9].

Extensive mitochondrial damage resulting from PA overload has been widely reported in electron microscopy and fluorescence microscopy studies [7]. These studies have highlighted the role of PA in mitochondrial fusion (indicative of a healthy cell) and fission (indicative of a cell under stress). A palmitic acid concentration above a certain threshold leads the cells towards death. Apoptosis is activated usually through the extensive release of reactive oxygen species (ROS) from a damaged mitochondrion [10]. ROS leakage then goes on to trigger apoptosis through cytochrome c and apoptosis-inducing factor (AIF) [11].

In this study, to obtain a better understanding of the whole process, we illustrated the mechanism through which PA overload affects endothelial cells by conducting an analysis of the differentially expressed genes (DEGs) observed in an excess of PA. Our major focus was to analyze the available dataset(s) to evaluate mitochondrial function-related genes and their impact on mitochondrial physiology, which then leads to pathology.

## 2. Materials and Methods

The raw datasets for the gene expression profile experiment of the palmitic acid (PA)-treated endothelial cell line were identified from the NCBI website. In total, 32 identified experiments were screened for eligibility. Following inclusion criteria assessment, the raw RNAseq dataset (GSE124522) of PA-treated human coronary artery endothelial cells (HCAEC) [12] was downloaded, normalized and analyzed to obtain differentially expressed genes (DEGs) using empirical analysis of differential gene expression in the R (EdgeR) package, with R software (version 4.1.3). Statistical significance was set at *p* < 0.05. Furthermore, mitochondrial-related functional genes were downloaded from the mitocarta database [13] and compared with the DEGs using the bioinformatics and evolutionary genomics online tool (http://bioinformatics.psb.ugent.be/webtools/Venn/, accessed on 30 April 2022). The overlapping genes between the DEGs and mitocarta genes were identified as mitochondria-related functional differentially expressed genes (mrfDEGs). Thus, they were considered in the subsequent analyses. 

**Figure 1:**
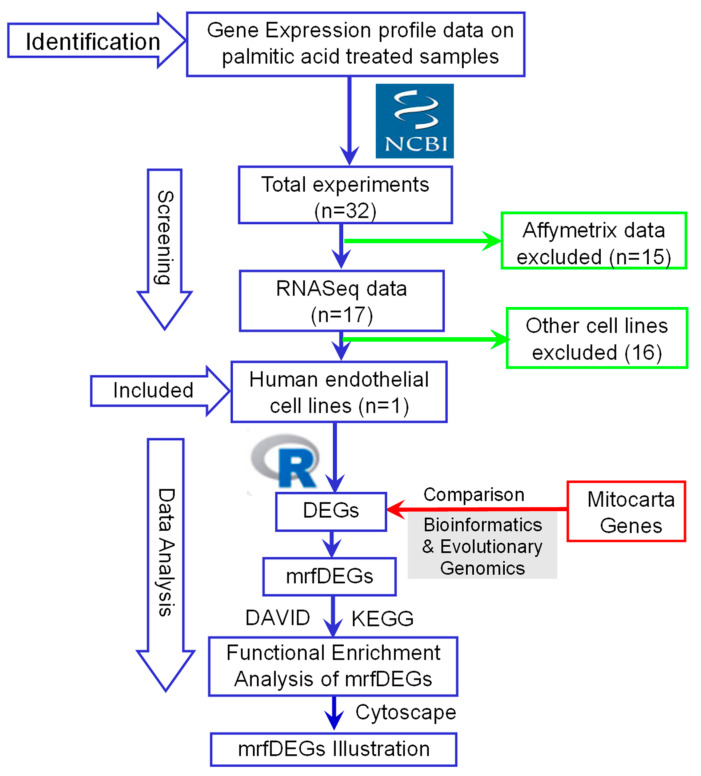
A flowchart describing the procedures used for the identification, selection, screening and analysis of data. DEGs: differentially expressed genes, mrfDEGs: mitochondrial-related functional DEGs, NCBI: National Center for Biotechnology Information.

To understand the biological role of the mrfDEGs, the Database for Annotation, Visualization and Integrated Discovery (DAVID) online tool (version 6.8) was used for the functional enrichment analysis [14,15]. mrfDEGs were subjected to DAVID for the analysis, using *Homo sapiens* as the reference species. The biological roles of the mrfDEGs were classified into three broad gene ontology categories: biological processes, molecular function and cellular compartments. Additionally, the Kyoto Encyclopedia of Genes and Genomes (KEGG) was used for pathway annotations of the mrfDEGs [16]. Protein–protein interaction (PPI) network analysis was performed with the aid of the STRING database [17]. Cytoscape (version 3.8.1) [18] was used to visualize the networks and patterns of expression of mrfDEGs. The procedure involved in the identification, selection, screening and analysis of data is summarized as a flowchart (Figure 1).

## 3. Results

**Figure 2:**
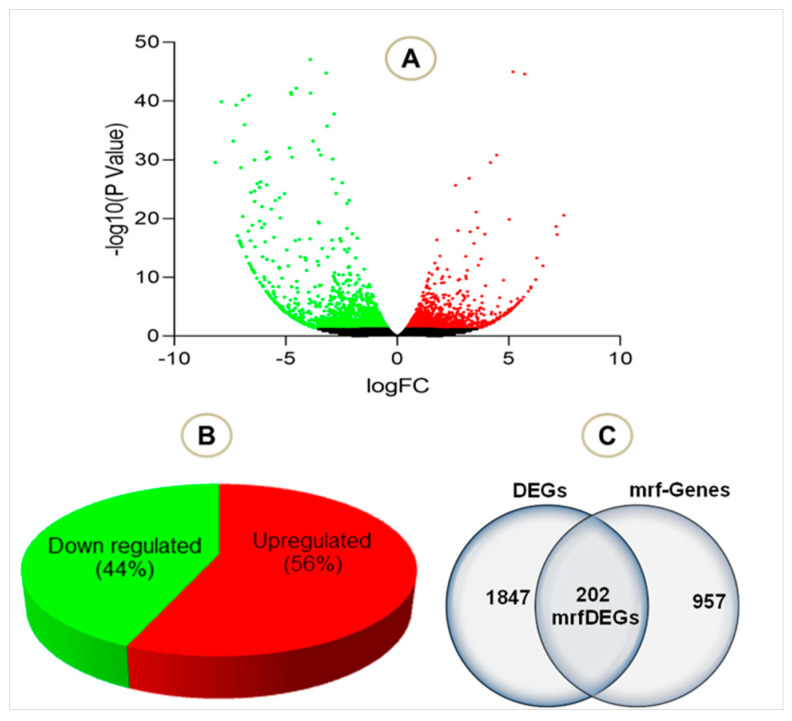
Total number of genes in the selected experiment (**A**) Volcano plot visualizing the differentially expressed genes (DEGs) in palmitic acid-treated human coronary artery endothelial cells (HCAECs). (**B**) Total number of downregulated and upregulated DEGs in percentage (**C**) Total DEGs, mitochondrial related functional genes and mrfDEGs. Green dot: downregulated genes, red dot: upregulated genes, black dot: not significantly regulated genes.

**Figure 3:**
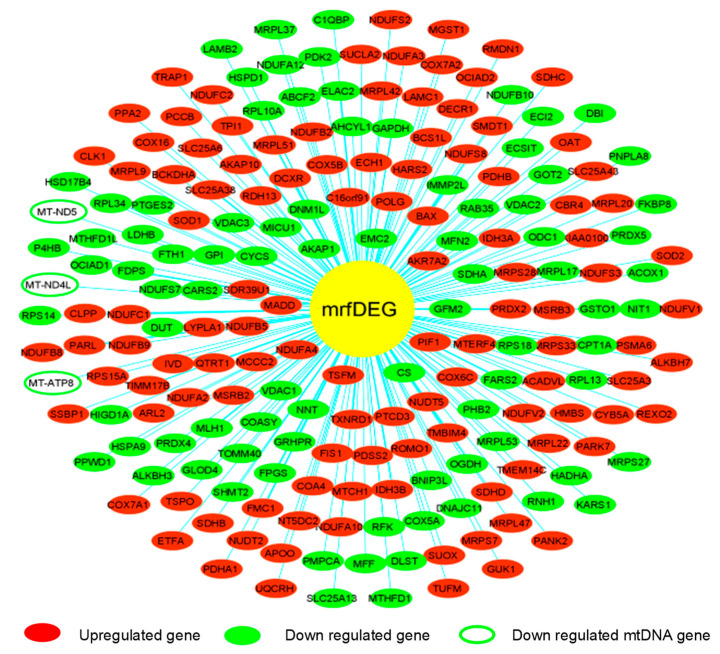
Pattern of expression of individual mitochondrial-related functional differentially expressed genes (mrfDEGs).

To evaluate the effect of PA treatment on mitochondrial genes, we extracted RNASeq raw data of endothelial cells treated with palmitic acid (PA) and dimethyl sulfoxide (DMSO) as a control treatment. The NCBI deposited experiment GSE124522 (sample ID: GSM3535804, GSM3535805, GSM3535806, GSM3535807, GSM3535816, GSM3535817, GSM3535818, and GSM3535819) fully satisfied our selection criteria; hence, it was considered for further analyses. To visualize the pattern of gene expression, volcanic plots were created (Figure 2A). The total 2047 genes were differentially expressed in HCAEC, treated with PA, where 56% genes were upregulated and 44% were downregulated (Figure 2B). Further analysis revealed that a total of 202 mrfDEGs were dysregulated due to the PA treatment (Figure 2C). In total, 957 mitochondrial-related functional genes were not present in our DEG list. The pattern of expression of individual mrfDEGs was viewed and illustrated (Figure 3). It was observed that 3 of the 13 protein-coding mtDNA genes, mitochondrially encoded NADH:Ubiquinone oxidoreductase subunit 5 (*MT-ND5*), mitochondrially encoded NADH:Ubiquinone oxidoreductase subunit 4L (*MT-ND4L*) and mitochondrially encoded ATP synthase membrane subunit 8 (*MT-ATP8*), were significantly downregulated (*p* < 0.05) in PA-treated HCAECs (Figure 3). 

**Figure 4:**
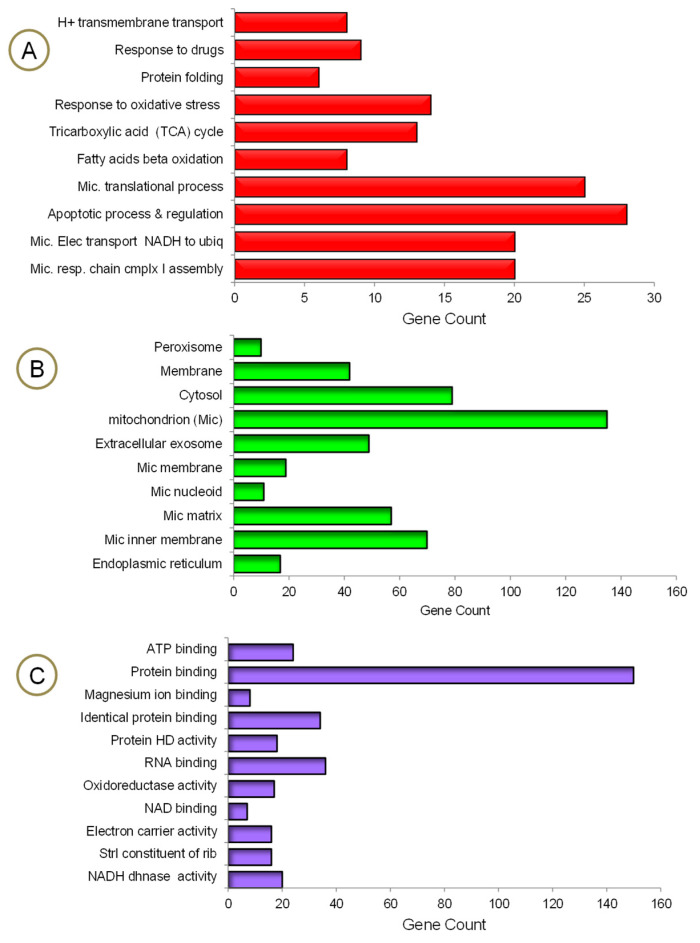
Gene ontology enrichment analysis of mrfDEGs. (**A**) Biological processes, (**B**) cellular compartments, (**C**) molecular functions. HD: homodimerization, Strl: structural, dhnase: dehydrogenease, rib: ribosome.

The mrfDEGs were subjected to DAVID for functional enrichment analysis to investigate the impact of PA treatment on mitochondrial and other cellular functions. The findings show that a significant number of genes required for many biological processes and molecular functions, operating exclusively in mitochondria, were altered (Figure 4). The process of obtaining energy from fatty acids has been jeopardized. This could be traced back to the number of mrfDEGs involved in fatty acid beta oxidation, the tricarboxylic acid cycle, H+ transmembrane transport, mitochondrial electron transport NADH to ubiquitin and mitochondrial respiratory chain complex 1 assembly (Figure 4A). Approximately 140 mrfDEGs were involved in the formation of mitochondria or performed important biological functions within them. Furthermore, a significant number of mrfDEGs were implied in structural formation or encoded proteins that played biological roles within the peroxisome, membrane, cytosol and/or endoplasmic reticulum (Figure 4B). In addition, it was observed that PA treatment had affected highly important molecular functions, including protein and nucleic acid binding, as well as enzymatic activities, e.g., oxidoreductase activity and NADH dehydrogenase activity (Figure 4C). Protein binding appeared to be the most affected molecular function owing to the large number of dysregulated genes involved in the process, and the NAD binding process remained the least affected molecular function. 

**Figure 5:**
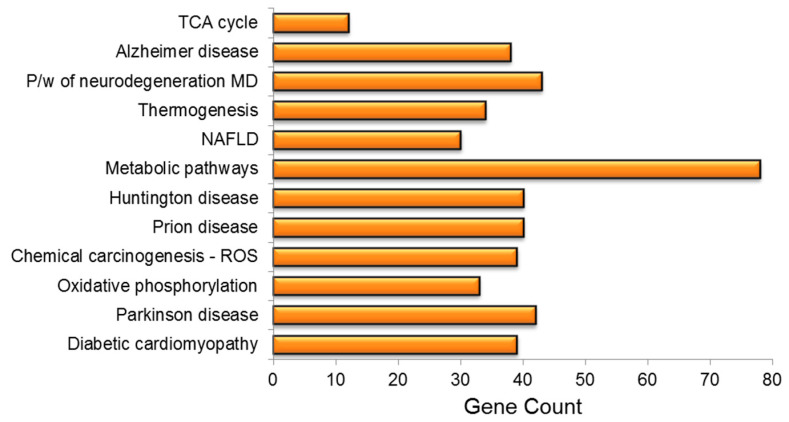
KEGG pathway enrichment analysis of mrfDEGs. TCA: tricarboxylic acid cycle, P/W: pathway, MD: multiple disease, NAFLD: non-alcoholic fatty liver disease, ROS: reactive oxygen species.

The KEGG pathway analysis was used to investigate the role of PA treatment in diseases and biological pathways in greater detail. The findings revealed that PA treatment had resulted in the dysregulation of vital genes linked to the development of neurodegenerative disorders, e.g., Alzheimer’s disease (AD), prion disease, non-alcoholic fatty liver disease (NAFLD), diabetic cardiomyopathy, Parkinson’s disease (PD), chemical carcinogenesis and amyotrophic lateral sclerosis, among others. PA treatment had a significant impact on several important pathways, including metabolic pathways, oxidative phosphorylation and thermogenesis (Figure 5).

**Figure 6:**
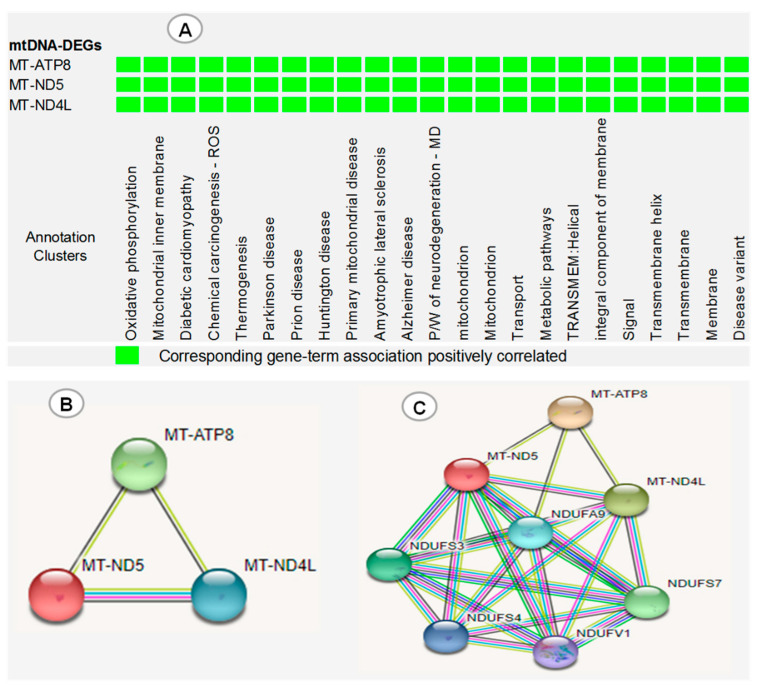
(**A**) DAVID functional annotation clustering of three dysregulated mtDNA DEGs (*MT-ND5, MT-NDL4* and *MT-ATP8*). (**B**) mtDNA DEG protein–protein interaction (PPI) network obtained by STRING analysis. (**C**) Expanded interaction showing the association of mtDNA DEGs with 5 closely related proteins of STRING database. ROS: reactive oxygen species, P/W: pathway, MD: multiple diseases. Red line: gene fusion, black line: gene co-expression, green line: evidence of gene neighborhood, blue line: evidence of co-occurrence, purple line: experimentally proven, yellow line: text mining.

The functional annotation clustering of three dysregulated mtDNA DEGs (*MT-ND5*, *MT-NDL4* and *MT-ATP8*) revealed that these genes were simultaneously involved in the development of many diseases, biological processes and important pathways (Figure 6A). Furthermore, STRING protein–protein interaction analysis showed that *MT-ND5*, *MT-NDL4* and *MT-ATP8* were co-expressed, evident from the black edge that connects one protein to the next, and have more interactions among themselves, with a PPI enrichment (*p* = 0.00000176) (Figure 6B). Thus, they have a partial biological relationship as a group. An expanded interaction performed to view the connections of mtDNA-DEGs with the five most closely related proteins in the STRING database revealed that MT-ND5 and MT-NDL4, but not MT-ATP8, have a similar and strong relationship with NADH:Ubiquinone oxidoreductase subunit A9 (*NDUFA9*), NADH:Ubiquinone oxidoreductase core subunit V1 (*NDUFV1*), NADH:Ubiquinone oxidoreductase core subunit S3 (*NDUFS3*), NADH:Ubiquinone oxidoreductase subunit S4 (*NDUFS4*) and NADH:Ubiquinone oxidoreductase core subunit S7 (*NDUFS7*), with a total edge of 24, and PPI enrichment (*p* = 0.0000000065) (Figure 6C).

## 4. Discussion

It is well known that free fatty acids (FFA), e.g., palmitic acid (PA), play an important role in many signaling processes, and an excess of it has a major impact on the physiology and functioning of mitochondria. This is manifested through a plethora of pathogeneses, diseases and disorders [6,7,8]. In the present study, we found DEGs in critical signaling pathways for cellular processes, e.g., apoptosis, protein binding, metabolic pathways and pathways involved in neurodegeneration. Out of 202 mitochondria-related functional genes (mrfDEGs), 13 mtDNA protein-coding genes were investigated; three of these mtDNA genes, *MT-ND5, MT-ND4L* and *MT-ATP8*, were significantly downregulated (*p* < 0.05) in PA-treated HCAECs. All these genes encode for proteins involved in oxidative metabolism (electron transport chain). Downregulation of these genes points towards specific processes, modulated in such a way that is not common in mitochondria. These findings indicated that PA induces a significant number of genes, thus providing insight into the pathways and networks related to mitochondrial function and metabolism. 

The second most significantly regulated set of genes were involved in metabolic pathways, neuronal diseases and cancer development. *NDUFA9*, *NDUFV1*, *NDUFS3*, *NDUFS4*, *NDUFS7* and *MT-ND5, MT-NDL4* were dysregulated genes with a strong correlation with each other. These genes play an important role in oxidative phosphorylation (OXPHOS) and ROS production. Another upregulated set of DEGs—superoxide dismutase type1 (*SOD1*), superoxide dismutase type2 (*SOD2*), methionine sulfoxide reductase B2 (*MSRB2*), microsomal glutathione transferase 1 (*MGST1*), peroxiredoxin 2 (*PRDX2*), peroxiredoxin 4 (*PRDX4*), methionine sulfoxide reductase B3 (*MSRB3*)—was also involved in ROS management in mitochondria and cells. The most important function of mitochondria is cellular respiration and metabolism, and almost all the above-mentioned dysregulated genes were involved in these two functions via the electron transport chain (mostly as subunits of different complexes of the electron transport chain). 

Mitochondria are well known for their role in cellular Ca^2+^ sequestering [19]. We observed that most of the genes in transmembrane transport, the mitochondrial inner membrane and the mitochondrial matrix were differentially regulated. Single-pass membrane protein with aspartate-rich tail 1 (*SMDT1)* was upregulated but mitochondrial calcium uptake 1 (*MICU1)* was downregulated. *SMDT1* is predicted to have a role in calcium import and mitochondrial calcium ion homeostasis, present in the mitochondrial inner membrane and nucleoplasm [20]. It is known as an important molecule for Ca^2+^ permeation and to regulate mitochondrial calcium uniporter (MCU) by binding to MICU using the conserved C-terminal poly-aspartate tail [21,22].

Voltage-dependent anion channel 1 (*VDAC1*), voltage-dependent anion channel 2 (*VDAC2*) and voltage-dependent anion channel 3 (*VDAC3*) were all downregulated. There are three known mammalian VDACs (VDAC1, VDAC2 and VDAC3) and they share a few functional and structural attributes. *VDAC1* is the most abundant isoform [22]; *VDAC2* knockout is lethal and it is known as an anti-apoptotic protein [23]. However, very little is known about *VDAC3* or how it is active as a channel [24]. Closure or downregulation of the *VDAC1* channel reduced the exchange of metabolites between the mitochondria and the rest of the cell, and inhibited cell growth [25]. This indicates its importance in the maintenance of physiological cellular function. VDAC1 plays an important role in the transfer of several other essential molecules, including Ca^2+^, cholesterol, fatty acids, ROS and ATP. VDAC1 controls the flow of Ca^2+^ to regulate mitochondrial Ca^2+^ homeostasis, oxidative phosphorylation and Ca^2+^ crosstalk between the *VDAC1* in the outer mitochondrial membrane and the inositol triphosphate 3 (*IP3*) receptor in the endoplasmic reticulum [26]. It takes place through the mitochondria-associated membranes (MAM), which mediate this exchange with the help of chaperone GRP75 [25,27]. ROS build-up leads to irreversible cysteine oxidation, which can maintain its open state. This open conformation can ultimately lead to the unregulated permeabilization of the mitochondrial outer membrane and, eventually, cell death [23,24,26]. BCL2 antagonist/killer (*Bak*) is an important regulator of apoptosis and was upregulated in the dataset. It has also been reported to interact with *VDAC*, which indicates increased Ca^2+^ sequestering and subsequent swelling of mitochondria, and possibly apoptosis [24]. 

One of the indicators of physiologically active and normally functioning cells is mitochondrial fusion, whereas mitochondrial fission indicates a cell moving towards apoptosis or cell death [26,28,29]. Mitochondrial fusion and fission are typically in a balance with one another; nutrient levels and other factors can create an imbalance and mitochondria can tip the balance towards either fusion or fission [30]. Our results indicated that two fusion genes, mitofusin 2 (*MFN2*) and mitochondrial fission factor (*MFF*), were downregulated, and one fission gene, fission mitochondrial 1 (*FIS1*), was upregulated. 

Mitochondrial dysfunction plays a substantial role in the imbalance of ROS and the antioxidant system in the cellular environment. ROS are well known to damage neurons and can accumulate in the brain, resulting in a host of neurodegenerative diseases [31]. Though metals are crucial for the enzyme-mediated reactions in cellular metabolism and cell signaling, mutation in mitochondrial DNA and metal overload in the aged brain subsequently lead to oxidative stress. A cascade of events causes the eventual impairment of neuronal proteins, resulting in neuroinflammation and neurological disorders manifested through the loss of cognitive function in Alzheimer’s disease (AD) [32], Parkinson’s disease (PD) [33], amyotrophic lateral sclerosis (ALS) [31] and Huntington’s disease (HD) [32]. Abnormal levels of ROS have also been widely linked to both metabolic syndrome (inflammation) and cancer development [34]. 

## 5. Conclusions

The major functions of mitochondria can be categorized into three broad categories: the electron transport chain (ROS production), mitochondrial membrane permeability and calcium sequestering. Differential expression of mitochondrial-related functional genes clearly shows their role in the proper operation of mitochondria, including the electron transport chain and Ca^2+^ sequestering. This has major implications for the functioning of mitochondria in endothelial cells in the presence of palmitic acid overload. This has also been extensively covered in the literature and linked to metabolic syndrome, neurodegenerative diseases, cancer and diabetes, among others.

## Data Availability

Not applicable.
